# The association between vitamin D and BDNF on cognition in older adults in Southern Brazil

**DOI:** 10.11606/s1518-8787.2022056004134

**Published:** 2022-12-13

**Authors:** Anna Quialheiro, Eleonora d´Orsi, Júlia Dubois Moreira, André Junqueira Xavier, Marco Aurélio Peres

**Affiliations:** I Universidade Federal de Santa Catarina Programa de Pós-Graduação em Saúde Coletiva Florianópolis SC Brasil Universidade Federal de Santa Catarina. Programa de Pós-Graduação em Saúde Coletiva. Florianópolis, SC, Brasil; II Universidade do Minho. Escola de Medicina Instituto de Investigação em Ciências da Vida e da Saúde Braga Portugal Universidade do Minho. Escola de Medicina. Instituto de Investigação em Ciências da Vida e da Saúde. Braga, Portugal; III Universidade Federal de Santa Catarina Programa de Pós-Graduação em Nutrição Florianópolis SC Brasil Universidade Federal de Santa Catarina. Programa de Pós-Graduação em Nutrição. Florianópolis, SC, Brasil; IV Universidade do Sul de Santa Catarina Palhoça SC Brasil Universidade do Sul de Santa Catarina. Curso de Medicina. Palhoça, SC, Brasil; V National Dental Research Institute Singapore National Dental Centre Singapore Singapore National Dental Research Institute Singapore. National Dental Centre Singapore. Singapore; VI Duke-NUS Medical School Oral Health ACP HealthServices and Systems Research Programme Singapore Duke-NUS Medical School. Oral Health ACP. HealthServices and Systems Research Programme. Singapore

**Keywords:** Aged, Cognition, Vitamin D, Brain-Derived Neurotrophic Factor

## Abstract

**OBJECTIVE:**

To estimate the association between vitamin D and the cognitive decline of older adults and evaluate whether this association is mediated by brain-derived neurotrophic factor (BDNF) serum concentration.

**METHODS:**

Cross-sectional study nested in a population-based cohort. Of the 604 participants in the complementary examination of the EpiFloripa Study, 576 older adults (60 years or older) were eligible for the study. The outcome is cognitive decline evaluated by the Mini-Mental State Examination, the exposure is vitamin D, and BDNF is the mediator. The control variables are age, sex, per capita family income, and educational level. The direct effect of vitamin D and BDNF on cognitive decline and the indirect effect mediated by BDNF was evaluated using path analysis, with the estimation of standardized coefficients.

**RESULTS:**

Among the participants, we observed a direct and positive effect of vitamin D on cognitive function (Coef: 0.06; 95%CI: 0.02 to 0.11; p < 0.001) and serum BDNF concentration (Coef: 21.55; 95%CI: 9.92 to 33.17; p = 0.002), i.e., the higher the vitamin D, the higher the cognitive function and serum level of BDNF.

**CONCLUSION:**

There was an association between vitamin D on serum BDNF and on cognitive decline in older adults. Moreover, BDNF did not have an effect on cognitive decline, so BDNF was not a mediator of the vitamin D effect on cognitive decline.

## INTRODUCTION

Age-associated cognitive decline can be a confounding factor to incipient dementia since it results in mental alterations, especially Alzheimer’s disease. The World Health Organization (WHO) considers dementia to be a public health priority since it is the leading cause of disability and dependency in older people worldwide^1^.

Vitamin D, also referred to as 25-hydroxy-D or 25(OH)D, has been widely studied as a neuroprotective factor^2^. The meta-analysis results showed that individuals with vitamin D deficiency have a 21% greater risk of developing Alzheimer’s disease than those with normal levels^3,4^. Vitamin D at insufficient levels (hypovitaminosis), defined as < 30 ng/mL, is described as a modifiable risk factor for dementia. In a systematic review on the relationship between vitamin D, cognition, and dementia, the authors reported that a concentration of less than 50 nmol/L of vitamin D is associated with a decline in cognitive function and a higher risk of Alzheimer’s disease^5^.

Brain-derived neurotrophic factor (BDNF) is a neurotrophin that plays a key role in inducing neuroplasticity, which promote the survival of neurons, and in modulating neuroplasticity in different interventions^6^. Moreover, BDNF plays an essential role in the central and peripheral nervous systems, especially in the hippocampus, favoring cognitive function during learning and memorization processes^7^. Little is known about the relationship between vitamin D and BDNF and its relevance in cognition. A population-based study on 300 Malaysian older adults identified that vitamin D derived from dietary intake, and not from supplementation, is a protective factor of cognitive function^8^. Another population-based study conducted in northern Germany analyzed the association between vitamin D and BDNF with depression and obesity and showed that non-obese individuals without depression had higher levels of vitamin D; however, no association with BDNF was found. The authors reinforced the role of vitamin D in mental health-related outcomes and suggested further studies to analyze this association with other health-related outcomes in the adult and older adult population^9^.

Cognition mainly involves the assessment of memory and learning, functions linked to the hippocampus, a region characterized by high concentrations of BDNF^10^.

Few studies were found regarding vitamin D and cognitive decline in the Brazilian population. According to the Brazilian Longitudinal Study of Aging (ELSI Study), the prevalence of vitamin D insufficiency in older adults was 17%, and the South region had the lowest serum concentration of vitamin D in the country^11^. Other studies show that vitamin D deficiency was highly prevalent in cognitive decline^12^, associated with diagnosed dementia, and it appears to be a marker of significant risk of functional decline. A systematic review with meta-analysis about nutritional strategies to manage Alzheimer’s disease showed that, in these patients, vitamin D supplementation had no significant effect on cognitive function or functional abilities^13^.

Within this context, a study on the association between vitamin D and BDNF on cognitive function in older adults is essential to prevent neurodegenerative diseases. Therefore, this study aims to estimate the association between vitamin D and cognitive decline of older adults and evaluate whether this association is mediated by BDNF serum concentration.

Thus, the following hypotheses were raised for this study: 1) there is an association between vitamin D and cognitive function; 2) there is an association between vitamin D and BDNF serum concentration; 3) there is an association between BDNF serum concentration and cognitive function, and therefore 4) BDNF serum concentration can be a mediator in the relationship between vitamin D and cognitive function.

## METHODS

This study is a cross-sectional analysis using data from the second wave of the EpiFloripa Aging Cohort Study, which included subjects aged 60 years or older living in the urban area of the city of Florianópolis, capital of the State of Santa Catarina, South Region of Brazil.

The baseline study (Wave 1) started in 2009–2010 and had two follow-ups: one in 2013–2015, called Wave 2; and another in 2017–2019, called Wave 3.

The study population was composed of older adults (60 years old or older) living in the urban region of Florianópolis in 2009–2010. The baseline sample size calculation was considered an expected prevalence of 50%, an error of four percentage points, and 95% confidence interval (95%CI), delineation effect (deff) for samples by clusters estimated as two. Clusters carried out the sample selection process in two stages. The first stage units were the census tracts (*Instituto Brasileiro de Geografia e Estatística* (IBGE) census units), and those in the second stage were the households. Further details of the study methodology can be found in previous studies^14^.

The sociodemographic and cognitive function data were collected using a pre-tested online questionnaire. After training, the interviewers went to the houses previously selected for data collection, where they conducted in-person interviews. The data consistency was verified weekly, and quality control was carried out by a telephone application of a reduced questionnaire to 10% of the participants, randomly selected by a supervisor^15^. Analysis of the reproducibility of the questions showed satisfactory to a good agreement (kappa of 0.5–0.9). During the interview, the subject was invited to attend a round of clinical tests at the university; upon accepting the invitation, a day, place, and time was appointed for sample collection, and the participants were given guidelines to be followed on the day of collection. They were oriented to fast 8–12 hours prior to the exam; in the examination room blood samples were collected for analysis in two steps. One aliquot was sent directly to the Clinical Analysis Laboratory of the University Hospital of the Federal University of Santa Catarina (UFSC) to analyze the lipid profile and vitamin D. Serum samples used to detect 25(OH)D were immediately processed using LIASON® 25 OH vitamin D assay (Diasorin, São Paulo, Brazil) accordingly to manufacture (Functional Sensitivity: ≤ 2.0 ng/mL; (inter-assay imprecision < 20%), which is considered a rapid, accurate, and precise assay^16^.

An aliquot of the remaining sample stored at -80ºC was used to measure BDNF by plate-based immunoassay (ELISA) at the Biochemistry Laboratory of UFSC. BDNF was analyzed using a commercial kit (DuoSet Human BDNF, DY248, R&D), and the plates were read in an M5 SpectraMax microplate reader (Molecular Devices, USA). The valid values followed the curve indicated in the commercial kit. Vitamin D was analyzed by microparticle chemiluminescence (Liaison method) in the same blood sample used to measure serum BDNF.

The serum concentration of 25-hydroxy vitamin D (25(OH)D; in ng/mL) was the exposure, analyzed as a continuous variable. The vitamin D levels were used as a categorical variable in the descriptive analysis according to Endocrine Society classifying in deficiency (under 20 ng/ml), insufficient (21 to 29 ng/ml), and normal level (30 ng/ml or higher)^17^.

The study’s outcome was the cognitive decline, which was assessed using the Mini-Mental State Examination (MMSE), validated for Brazil^18^. The MMSE is used to evaluate cognitive functions on a scale from 0 to 30, and the classification is made based on educational level, with older adults being classified as having possible cognitive impairment when they achieve a score < 19 (illiterate) or < 23 (formal education). Cognitive decline was used as a dichotomous variable in the descriptive analysis according to Almeida^18^ (1998), and as a continuous variable in path analysis.

The serum BDNF concentration (in pg/mL) was also the mediator variable, analyzed as a continuous variable. The adjustment variables collected by interview were sex (man or woman); age group (60–64, 65–69, 70–74, 75–79, and ≥ 80 years); per capita family income in Brazilian minimum wage (≤ 1, 1–5, 5–10, and ≥ 10), educational level in complete years of schooling classified as formal education (no formal education, 0; 1–4 years; 5–8 year; 9–11 years; ≥ 12 years).

This study was approved by the Ethics Committee on Research Involving Humans of Federal University of Santa Catarina (Approval No. 16731313.0.0000.0121) in 2013, and all participants signed a free and informed consent form.

### Statistical Analysis

Age, family income, educational level, and vitamin D (ordinal polytomous variables) were compared between groups using one-way ANOVA. Descriptive statistics with absolute and relative frequencies were used to describe the sociodemographic profile of the participants by vitamin D and BDNF serum concentration. A t-test for independent samples was applied to determine whether the groups were comparable regarding sex and cognitive impairment (dichotomous variables). Differences were considered significant when p < 0.05.

**Figure 1:**
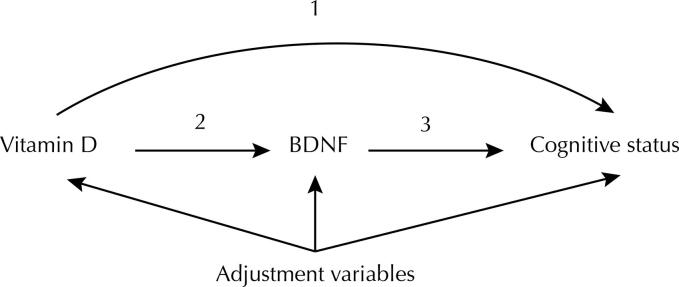
Directed acyclic graph, including the variables studied. (1) Direct effect of vitamin D on cognitive decline. (2) Direct effect of vitamin D on brain-derived neurotrophic factor (BDNF). (3) Direct effect of BDNF on cognitive decline. (2 + 3) Indirect effect of vitamin D on cognitive decline mediated by BDNF. The adjustment variables were age, sex, educational level, body mass index, and physical activity.

The relationship between variables by pathway was illustrated using Directed Acyclic Graphs (DAG). These graphs are a rapid and visual method to identify the effect of an exposure variable on the outcome and possible confounding variables. In the DAG of this study, vitamin D was the exposure, cognitive decline was the primary outcome, and sex, age, family income, and educational level were the adjustment variables. In this study, BDNF was considered a secondary outcome and the mediator of the pathway between vitamin D and cognitive decline (Figure 1).

To analyze the associations between vitamin D, BDNF, and cognitive decline, path analysis was used as an alternative approach to the traditional method for testing mediating effects and multivariate analysis of direct and indirect effects. Path analysis analyzes the mediating effect due to better power and more accurate type I error rates^19^. Thus, the path analysis was conducted to estimate the pathways among the main variables to analyze the effect of the vitamin D on (1) cognitive score and (2) BDNF serum levels. Additionally, indirect effects of BDNF on the relationship between vitamin D and cognitive score were analyzed through path analysis.

The standardized coefficients adjusted for sex, age, family income, and educational level, and their respective 95% confidence intervals, were calculated with the IBM Stata 14.0 software, adopting a level of significance of 5%.

The dataset was published in Mendeley Data and should be requested to the corresponding author, and it is available at http://dx.doi.org/10.17632/xfzkhn94dr.1.

## RESULTS

**Figure 2:**
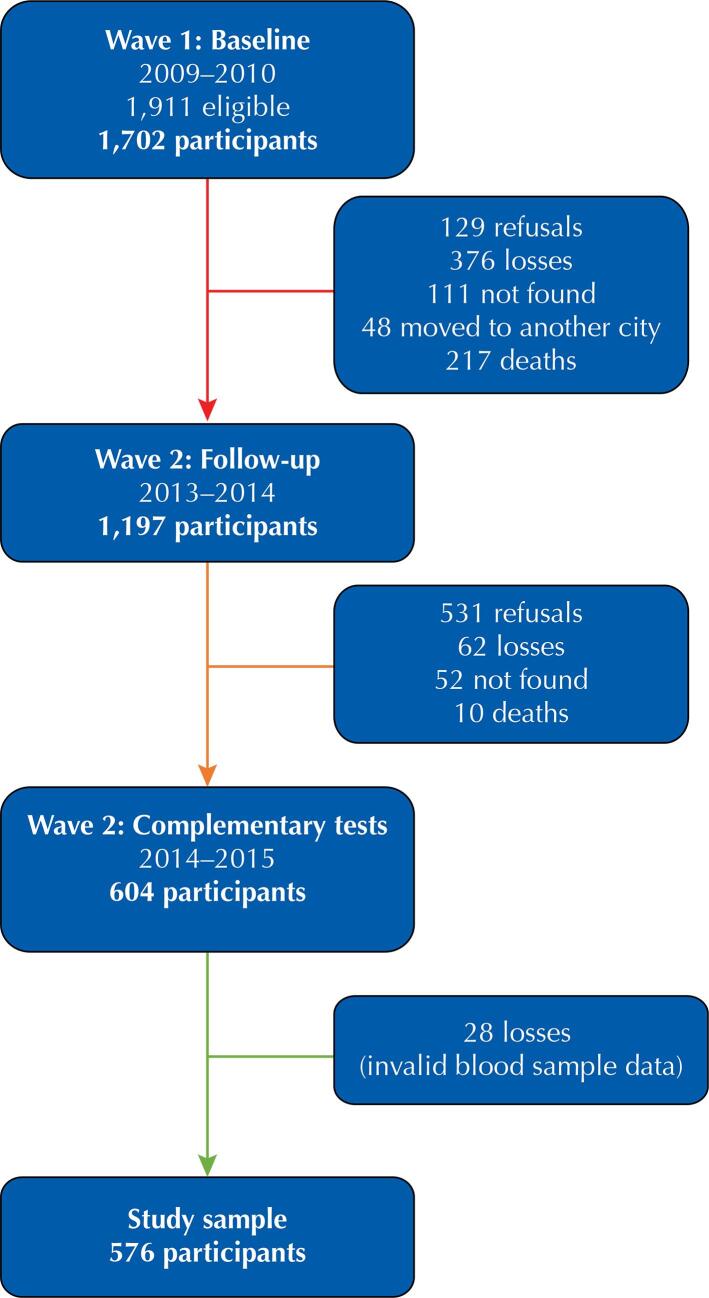
Waves of the EpiFloripa Ageing Study, Wave 1 and 2.

A total of 576 older adults participated in the study, corresponding to 48.1% of the eligible sample of Wave 2 (n = 1,197). There were 44.4% refusals (n = 531); 4.3% losses, since the subjects could not be located for scheduling the examination (n = 52); and 0,8% of deaths (n = 10), resulting in 604 older adults with a blood sample. Of those 604, there were 2.3% losses due to invalid values for vitamin D and BDNF (n = 28), resulting in 576 older adults for study analysis (Figure 2).

The mean age of the participants (n = 576) was 72.4 years (range 63–93 years); 65.2% were women, 42.6% had less than four years of schooling, 57,1% had a family income between 1 and 5 Brazilian minimal wage. Approximately one-fifth of the participants had cognitive impairment, and 26.4% had vitamin D insufficiency, i.e., levels < 30 ng/mL, according to the Endocrine Society^17^.

Women had, on average, significantly lower vitamin D levels than men (25.2 *versus* 28.6 ng/mL). There was no difference in vitamin D with increasing age, family income, or education levels. Thus, older adults with cognitive impairment had a significantly lower average of vitamin D levels than older adults without cognitive impairment (Table 1).

**Table 1:** Association of sociodemographic with vitamin D and serum BDNF concentration in the older adults, Brazil, 2019.

	Maximum sample (n = 576)		Vitamin D (ng/ml)		BDNF (pg/ml)	
	n	%	Mean ± SD	p^a^	Mean ± SD	p^a^
Sex				**< 0.001**^d^		0.465
Women	375	65.1	25.2 ± 8.2		3,316.5 ± 1,264.9	
Men	201	34.9	28.6 ± 10.3		3,233.1 ± 1,333.8	
Age (years)				0.056		0.501
60–69	244	42.4	27.1 ± 9.5		3,381.5 ± 1,387.2	
70–79	239	41.5	26.5 ± 8.4		3,206.7 ± 1,233.2	
80–89	88	15.3	24.3 ± 9.8		3,229.8 ± 1,148.1	
≥ 90	5	0.8	20.8 ± 8.9		3,318.4 ± 1,003.0	
Family income (Brazilian minimal wage)^b^				0.105		0.339
< 1	147	26.4	25.6 ± 7.9		3,235.8 ± 1,304.5	
1–5	318	57.1	26.4 ± 9.9		3,363.7 ± 1,297.1	
5–10	64	11.5	28.7 ± 8.6		3,225.1 ± 1,262.1	
≥ 10	28	5.0	28.0 ± 6.9		2,936.6 ± 1,183.3	
Education (years)				0.130		0.431
0–4	245	42.6	26.1 ± 9.8		3,213.6 ± 1,236.7	
5–8	104	18.1	25.4 ± 8.5		3,330.6 ± 1,313.5	
9–11	87	15.1	26.0 ± 8.8		3,471.4 ± 1,384.8	
≥ 12	139	24.2	27.4 ± 8.4		3,253.3 ± 1,297.4	
Cognitive function^c^				**0.013**^d^		0.582
With cognitive deficit	124	21.5	24.6 ± 8.8		3,227.2 ± 1,258.4	
Without cognitive deficit	448	78.5	27.0 ± 9.2		3,299.9 ± 1,299.5	
Vitamin D				-		**0.007**^d^
Deficiency	152	26.4	15.5 ± 4.1		3,038.0 ± 1,120.9	
Insufficiency	231	40.1	25.6 ± 2.6		3,288.9 ± 1,322.2	
Normal	193	33.5	36.0 ± 6.4		3,486.3 ± 1,333.7	

^a^ The data was analyzed with a T-test for independent samples (dichotomous variables) and one-way ANOVA (polytomous variables).

^b^ Minimum sample is 557.

^c^ Classification based on Almeida^18^. ^d^ p < 0.05.

SD: standard deviation; BDNF: brain-derived neurotrophic factor.

95%CI: 95% confidence interval; BDNF: brain-derived neurotrophic factor.

**Figure 3:**
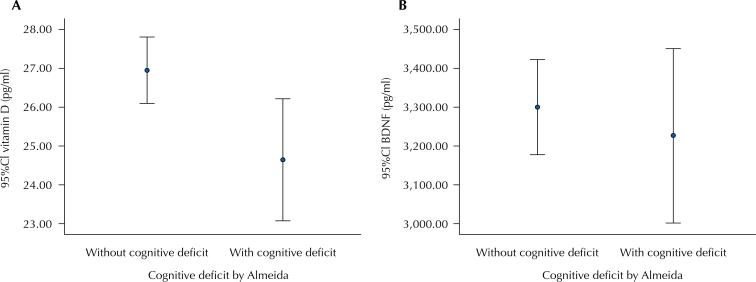
(A) vitamin D levels and (B) BDNF levels according to cognitive decline.

No significant differences in BDNF concentration were observed when sex, age, family income, educational level, or cognitive decline were compared. On the other hand, there were a significant increase in the mean BDNF serum concentration between individuals with deficient (≤ 20 ng/mL), insufficient (21–29 ng/mL), and normal vitamin D levels (≥ 30 ng/mL) (Figure 3).

An association between vitamin D and cognitive decline (Coef: 0.06; 95%CI: 0.02–0.11; p < 0.001) was observed, i.e., for each 10 ng/ml increase of vitamin D, the MMSE scores increased by 0.6 points . We observed an association between vitamin D and BDNF serum concentration (Coef: 22.55; 95%CI: 9.92–33.17; p = 0.002), i.e., for each 10 ng/ml increase of vitamin D, the BDNF increased by 225.5 pg/ml. This association was significant in crude and adjusted analysis by sex, age, family income, and educational level (Table 2).

**Table 2:** Multivariate model for the association between vitamin D and cognitive decline mediated by BDNF serum concentration in older adults.

Path (n = 529)	Coef.	Crude analysis			Adjusted analysis^a^			
		95%CI		p	Coef.	95%CI		p^a^
Vitamin D → Cog	0.06	0.02	0.11	**< 0.001**^b^	0.04	0.001	0.007	**0.040**^b^
Vitamin D → BDNF	21.55	9.92	33.17	**0.002**^b^	23.09	11.1	35.1	**< 0.001**^b^
BDNF → Cog	-0.000	-0.000	0.000	0.840	-0.000	-0.000	0.000	0.798
Vitamin D → BDNF → Cog	-0.000	-0.007	0.006	0.840	-0.001	-0.013	0.011	0.917

^a^ Pathway analysis adjusts by sex, age, family income, and education.

^b^ p < 0.05

95%CI: 95% confidence interval; BDNF: brain-derived neurotrophic factor.

There was no association between BDNF and cognitive decline, so serum BDNF concentration did not mediate the relationship between vitamin D and cognitive decline (Table 2).

## DISCUSSION

This study found a direct and positive association of vitamin D on cognitive function and serum BDNF concentration, i.e., the higher the vitamin D level, the higher the cognitive function and the serum level of BDNF. There was no effect of BDNF on cognitive decline, so BDNF was not a mediator of the effect of vitamin D on cognitive decline.

The association between vitamin D and cognitive decline and BDNF serum concentration found in this study may indicate the importance of improving vitamin D levels, with supplementation or sun exposure, as a strategy for preventing and treating cognitive decline and increasing BDNF serum concentration in older adults^20^. A study investigating neuroprotective factors for successful aging in Malaysian older adults showed that higher vitamin D and BDNF serum concentration are associated with successful aging; however, the study did not analyze the effect of vitamin D on BDNF. That study defined successful aging when the individuals had no arterial hypertension, diabetes, cardiovascular and pulmonary diseases, had a good quality of life and health perception, good overall cognitive function indicated by an MMSE score higher than 22, fewer than five depressive symptoms by the Geriatric Depressive Scale, and no functional limitation in activities of daily living^8^.

The Yosefian et al.^21^ study analyzed the effect of vitamin D on BDNF concentration with an animal model to investigate the use of vitamin D in treating depression, and the results showed that vitamin D did not affect BDNF concentration in the hippocampus. A study by Xu et al.^22^, also on depression and with an animal model, showed that the modulation of hippocampal BDNF by vitamin D treatment could be an effective strategy for preventing and treating post-stroke depression. The measurement of vitamin D and BDNF was within the hippocampus, and the results showed that the increase in vitamin D increased BDNF levels.

Despite the conflicting results found in the literature, experimental studies have pointed to the importance of vitamin D on hippocampal neurodevelopment during gestation and in the early stages of brain development after birth, and restoring vitamin D levels may represent a helpful strategy for healthy brain aging^2,23^.

According to a systematic review about the effect of low vitamin D on cognition, studies have presented different neuropsychological assessment methods. Most of the studies used MMSE to evaluate general cognition, whereas other studies used subdomains as executive function, verbal fluency, and verbal episodic memory. Evidence suggests psychomotor and executive functions are most susceptible to fluctuations in vitamin D physiology during aging. It is crucial to standardize methods and coverage of cognitive domains to draw firm conclusions on the differential effects of vitamin D on specific cognitive abilities. The mechanisms by which vitamin D modulates cognitive processes in aging and the neuro-pathophysiology of dementia are complex. Although vitamin D has been shown to elicit neuroprotective properties via calcium homeostasis and maintaining the integrity of nerve conduction, only observational studies indicated a true effect of vitamin D on cognition^24^.

There was no significant difference in the vitamin D and BDNF serum concentration between the compared groups analysis across categories of age, family income, or educational level. Regarding vitamin D and cognitive decline, higher vitamin D was observed in older adults without cognitive deficit. The literature suggests a concern with an increase in cognitive deficit, since the prevalence of hypovitaminosis D (deficiency and insufficiency) is increasing worldwide. Studies on older adults show that vitamin D deficiency increases the risk of loss in MMSE score^20,25^. Another study compared mean MMSE scores and found higher vitamin D levels in those with a higher test score^3^. Systematic reviews with meta-analysis demonstrated that a low vitamin D concentration is associated with a decline in cognitive function and an increased risk of Alzheimer’s disease^3,4^.

On the other hand, this study did not show the association between BDNF, a neuroplasticity factor, and cognitive decline or BDNF as a mediator between vitamin D and cognitive decline. Although studies investigating BDNF and cognitive function demonstrated that a reduction in the levels of this marker is associated with cognitive decline^26,27^, we believe that adaptive brain and body responses mediated by BDNF influence aren’t clear in the literature.

The relationship between physical activity, serum BDNF concentration, and cognitive function has been extensively studied, and the results show that acute or vigorous exercise increases peripheral BDNF concentration, with positive effects on cognition^28,29^. In our study, older adults who were physically active, i.e., performed more than 150 minutes of activity per week, had higher peripheral BDNF concentration. However, one study found that long-term habitual exercise is associated with lower peripheral BDNF in more active individuals, despite showing an improvement in memory^29^, one of the fundamental domains of cognitive function. An intervention study with mediation analysis involving individuals aged 55 to 80 years showed that serum BDNF concentration mediates the effects of walking on improvements in cognitive function as a function of age. However, no significant differences between the intervention and control groups were reported.

Although several studies show that physical activity increases BDNF, a systematic review showed that the significant increase in BDNF depends on the modality and intensity of the exercise. Eight studies examining BDNF changes were suited for metanalysis and showed that higher BDNF concentrations were reached post-intervention, but did not reach statistical significance^30^.

### Strengths and Limitations of the Study

The EpiFloripa Ageing Cohort Study has investigated older adults in three waves, with a response rate of 70.3% between Wave 1 and Wave 2, and 79.8% between Wave 2 and Wave 3. In this study, we used a population-based sample with a high response rate of more than 70% in the first two waves considering the age range of the study. The study was conducted by a team experienced in cohort studies, and internationally validated instruments were used. Additionally, statistical analysis permitted the correct identification of direct and mediated effects.

On the other hand, to the best of our knowledge there is no available literature on the reference values for serum BDNF concentration in older adults. Studies only show serum levels of this marker comparing its concentrations before and after an intervention or evaluating its association with cognitive outcomes^31^. For example, a study conducted in Brazil with 143 older adults reported high levels of BDNF serum concentration, above the fourth quartile (> 2,378.4 pg/mL) of BDNF concentration of the control group, in individuals with mild cognitive impairment or Alzheimer’s disease^32^. Another study carried out in Ukraine with 59 older adults showed an increase in BDNF plasm concentration after drug treatment for comorbidities related to cognitive impairment (20,660.4 to 26,356.0 pg/ml)^33^. The values found in the studies with serum BDNF have a widely varied range.

Another limitation of this study is that it is a cross-sectional analysis, which limits the interpretation of the data. The loss of follow-up should also be considered a limitation since the data collection for this cohort was conducted via at-home interviews. The collection of blood data can only be carried out with the participants’ displacement to a collection center. Despite the 50.9% adherence of eligible older adults for blood collection in Wave 2, the number of participants remained relevant (n = 604) considering human studies with blood collection outside the home. The EpiFloripa Ageing Study has a Wave 3 with cognitive decline evaluation. A sensitive analysis was performed to verify the variation of the vitamin D and BDNF with cognitive decline in Wave 3. The results show that vitamin D had no direct effect on cognitive decline and BDNF had no significant effect on cognitive decline (Table 3).

**Table 3:** Multivariate model for the association between vitamin D and cognitive decline on the next wave, mediated by BDNF serum concentration in older adults.

Path (n = 529)	Coef.	Crude analysis			Adjusted analysis^a^			
		95%CI		p	Coef.	95%CI		p^a^
Vitamin D → Cog	0.03	-0.02	0.08	0.187	0.01	-0.03	0.05	0.523
Vitamin D → BDNF	22.09	7.6	36.6	**0.003**^b^	21.91	7.02	36.79	**0.004**^b^
BDNF → Cog	-0.000	-0.000	0.000	0.778	-0.000	-0.000	0.000	0.623
Vitamin D → BDNF → Cog	-0.001	-0.008	0.006	0.779	-0.001	-0.008	0.006	0.623

^a^ Pathway analysis adjusts by sex, age, educational level, body mass index, and physical activity

^b^ p-value < 0.05.

95%CI: 95% confidence interval; BDNF: brain-derived neurotrophic factor; Cog: cognitive.

This study shows an association between vitamin D on serum BDNF and vitamin D on cognitive decline in older adults. These results show the importance of increased vitamin D levels to improve mental health, especially related to cognition.

Regarding BDNF serum levels, this study shows no association between BDNF and cognitive scores. Since the literature presents studies in which higher serum BDNF is related to better mental health, the authors believe that investigations are still needed to confirm this evidence. However, further well-designed, longitudinal, and randomized controlled trials are required to assess the effects of vitamin D and BDNF on cognition.
